# Virtual reality‐based therapy after anterior cruciate ligament injury effectively reduces pain and improves knee function, movement patterns, and dynamic balance: A systematic review and meta‐analysis

**DOI:** 10.1002/ksa.12477

**Published:** 2024-09-20

**Authors:** Irene Cortés‐Pérez, Jose María Desdentado‐Guillem, María Soledad Camacho‐Delgado, María del Rocío Ibancos‐Losada, Esteban Obrero‐Gaitán, Rafael Lomas‐Vega

**Affiliations:** ^1^ Department of Health Sciences University of Jaén Jaén Spain

**Keywords:** anterior cruciate ligament injuries, muscle strength, musculoskeletal pain, postural balance, rehabilitation, virtual reality exposure therapy

## Abstract

**Purpose:**

Virtual reality‐based therapy (VRBT) may be an effective physical therapy complement employed in the rehabilitation of patients with anterior cruciate ligament (ACL) injury. This study aims to assess the effectiveness of VRBT in improving pain, knee function, strength, proprioception, flexion range of motion (ROM), and dynamic balance after ACL injury.

**Methods:**

We conducted this systematic review with meta‐analysis following PRISMA criteria. Since inception to June 2024, we searched in PubMed Medline, WOS, SCOPUS, CINAHL and PEDro without publication date and language restrictions. Randomised controlled trials (RCTs), comprising only patients with ACL injury, that assess the effectiveness of VRBT compared to classical interventions on the outcomes of interest were included. PEDro scale was employed to analyze the methodological quality of the RCTs included. Cohen's standardised mean difference (SMD) and its 95% confidence interval (95% CI) was used to calculate the pooled effect in meta‐analyses.

**Results:**

Nine RCTs, providing data from 330 participants (26.96 ± 3.11 years, 85% males) were included. The RCTs included showed good methodological quality (PEDro scale = 6.88 points), being, performance and detection biases, the most common biases reported. Meta‐analyses showed that VRBT was more effective than classical interventions in reducing pain (SMD = −1.15; 95% CI −1.85 to −0.45; p = 0.001; *I* ^2^ = 0%), and increasing knee function (SMD = 1.71; 95% CI 0.93 to 2.5; *p* < 0.001; I^2^ = 0%), strength (SMD = 0.82; 95% CI 0.4–1.23; *p* < 0.001; *I* ^2^ = 0%) and flexion ROM (SMD = 0.7; 95% CI 0.37–1.01; *p* < 0.001; *I* ^2^ = 0%). Additionally, VRBT improved postero‐medial (SMD = 0.46; 95% CI 0.01–0.9; *p* = 0.045; *I* ^2^ = 15.1%) and postero‐lateral CoP excursion (SMD = 0.75; 95% CI 0.3–1.21; *p* = 0.001; *I* ^2^ = 0%), being effective in improving dynamic balance.

**Conclusion:**

VRBT is an effective physical therapy complement to be included in the ACL's rehabilitation programmes due to reduces pain and increases knee function, strength, ROM and dynamic balance after ACL injury.

**Level of Evidence:**

Level II.

## Graphical abstract

Effectiveness of virtual reality‐based therapy in the rehabilitation of the anterior cruciate ligament injury. A systematic review and meta‐analysis.

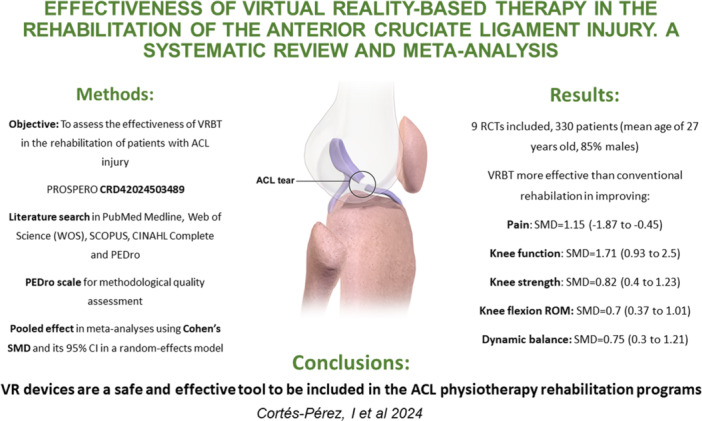

**Abbreviations**
ACL: anterior cruciate ligament
RCTs: randomised controlled trials
ROM: range of motion
SMD: standardised mean difference
VR: virtual reality
VRBT: virtual reality‐based therapy

## INTRODUCTION

Anterior cruciate ligament (ACL) injury represents a serious injury to the knee joint [29], mainly affecting a lot of elite athletes every year [56]. Indirect contact with dynamic knee valgus loading, shallow knee flexion and ipsilateral trunk tilt [39, 53, 62] is the main cause of ACL injuries in sports (88% of cases) [80], although others secondary risk factors are lack of strength and poor neuromuscular control, increased Q angle, and female sex (eight times higher than in male) [47]. According to the consensus of the German Knee Society, surgery continues as the gold standard intervention for treating ACL injury [29, 61, 62]. Rehabilitation plays a key role in improving knee functional outcomes (pain, range of motion [ROM], strength, proprioception or balance [44, 74]), helping athletes regain their pre‐ACL injury performance level [19]. Conservative rehabilitation, which includes physiotherapeutic measures [62], aimed at enhancing muscle strength, ROM, proprioception, and joint stability, is essential for improving knee function and balance [52, 81] both before and after ACL surgery. This approach is crucial for reducing postoperative complications and ensuring surgical success [64]. Additionally, it may serve as an alternative to surgery in cases of ACL injuries or conditions where surgical intervention is not immediately indicated [50, 62]. Previous studies have showed that psychological factors also play a key role in rehabilitation [53], and monotonous feeling experienced by patients during classical physiotherapy interventions can reduce the self‐efficacy and motivation. Therefore, it is necessary to search novel approaches in rehabilitation that reinforce the commitment and adherence of the patient to their rehabilitation.

In recent years, the use of virtual reality (VR) devices in knee injuries rehabilitation has increased [25]. VR devices present two fundamental characteristics: presence (feeling to perceive the virtual environment as real) and immersion (capability to interact with this virtual environment) [55]. In non‐immersive VR (NIVR) these level of presence and immersion is reduced and patients visualise virtual bi‐dimensional environments projected in screens and with which the subject interacts using a mouse, keyboard and joysticks [70]. Opposite, immersive VR (IVR) uses head‐mounted displays that incorporate surround sound to maximise immersion and allow three‐dimensional visualisation of the virtual environment in 360° [59]. Due to their low cost, difficulty to use and adverse events, NIVR devices are the most used in rehabilitation. VR devices uses gamified software that can be commercial video games, exergames or serious games. Serious games use the characteristics of commercial video games to train, and exergames combine elements of game‐based learning with different ways of engaging in physical activity [46]. VR devices with gamified exercises allow to improve the performance of motor functional tasks in funny and ludic environments that increase the motivation and commitment of the patients with their rehabilitation process [60, 63].

To date, several reviews have assessed the effectiveness of virtual reality‐based therapy (VRBT) in the rehabilitation of different knee injuries with promising results [24, 57, 77]. Only one systematic review with meta‐analysis, including five studies and 128 patients, have assessed the effect of exergames (VR devices and smartphone app games) after ACL injury [21]. The main variables assessed in that review were pain, proprioception and strength with data from two studies each one, and other secondary variables, with data from only study were knee function and balance. Fernandes et al., [21] did not show differences between exergames and others interventions for pain, knee strength and ROM, function, and balance. It is necessary to update the literature search in comparison to previous review, due to it was carried out since inception up to June 2021, and new studies published, providing data from more participants can vary the previous findings, increasing the level of evidence. Finally, RCTs and control‐case studies were included in the previous reviews, reducing the level of evidence, and to homogenise the findings, it is necessary to establish new inclusion criteria with only RCT studies (without healthy subjects). Therefore, the aim of our systematic review with meta‐analysis is to compile all related studies to date to analyze the effect of VRBT in the rehabilitation of ACL injury.

## MATERIALS AND METHODS

### Design and protocol review

To carry out this systematic review with meta‐analysis, the recommendations of the PRISMA (Preferred Reporting Items for Systematic Reviews and Meta‐Analyses 2020 version) and the Cochrane Handbook of Systematic Reviews of Interventions, were followed and the [31, 58]. This systematic review was appraisal using the AMSTAR 2 checklist [72]. Furthermore, this systematic review was previously registered in the PROSPERO database.

### Literature search and sources

Two authors (blinding for peer review), independently, carried out a bibliographic search in PubMed Medline, Web of Science (WOS), SCOPUS, CINAHL Complete and PEDro (Physiotherapy Evidence Database) since inception up to June 2024. Additionally, authors screened the reference lists of the related reviews, potential studies to be included and others sources (study registries, documents from experts in the field and grey literature). The search strategy was developed following the PICOS system: [2] population (patients with ACL injury), intervention (VRBT), comparison (conventional therapy intervention), outcome (pain, knee function, strength, proprioception and flexion, and postural balance) and study design (randomised controlled trials [RCTs] or pilot RCTs). Authors decide to perform a sensitive search using alone the population and interventions dimensions joined with AND, and the related term in each dimension joined with OR. The keywords used in the search were selected from terms indexed in the PubMed thesaurus (MeSH [Medical Subject Headings]): virtual reality, virtual reality exposure therapy, exergaming, anterior cruciate ligament, anterior cruciate ligament reconstruction and anterior cruciate ligament injuries, and entry terms. No limitations were imposed on language or publication date. Finally, a third author (blinding for peer review) validated the search carried out in the different databases through the peer review process. Supporting Information: Table 1 shows in detail the search strategy used in each database.

### Study selection: inclusion and exclusion criteria

Two authors (blinding for peer review), independently, analysed each retrieved study by title and abstract and the level of agreement about the inclusion studies was assessed using the Cohen's kappa coefficient (*κ*) [8], that can be interpreted as *κ* < 0 (non‐existent); 0 ≤ *κ* ≤ 0.2 (non‐significant); 0.2 < *κ* ≤ 0.4 (discrete); 0.4 < *κ* ≤ 0.6 (moderate); 0.6 < *κ* ≤ 0.8 (substantial); 0.8 < *κ* ≤ 1 (excellent) [40]. Disagreements were arbitrated by a third author (blinding for peer review). The inclusion criteria were the followings: (1) RCT or pilot RCT; (2) studies carried out on patients with ACL injury; (3) in which the intervention group received VRBT (isolated or combined with other therapies); (4) compared to a control group that performed any conventional physiotherapy intervention; (5,6) that included quantitative data of the variables of interest to perform the meta‐analysis. RCTs that included patients with ACL injury with others knee pathologies in the same group; and (2) studies with a comparison group comprised by others knee injuries, were the exclusion criteria followed.

### Data extraction

All data extracted from the selected studies were collected in a Microsoft Office Excel data collection form by two authors (blinding for peer review). Finally, the data were confirmed through review by a third author (RLV). First, general characteristics of each RCT included were extracted (authors, publication date, country, setting, funding and blinding). Second, we compiled the general characteristics of the sample (number of participants, participants per group, age, biological sex and time since diagnosis); and the characteristics of the intervention group (type of VR used, treatment protocol [number of sessions, duration of sessions, sessions per week and minutes per session] and the control group (type of conventional physiotherapy used and treatment protocol). Finally, related with variables, we extracted the name of the variable, the measurements employed, the statistical data to perform the meta‐analysis (participants per group, mean and standard deviation (post‐intervention), and the time‐point of evaluation.

### Variables

The outcomes examined in the studies in the review were as follows: pain, knee function, knee strength, knee proprioception, knee flexion ROM and dynamic balance.

### Assessment of methodological quality, risk of bias and quality of evidence

Methodological quality and, secondarily risk of bias, were assessed using the PEDro scale by two authors (blinding for peer review). This scale is an evaluation method designed to measure the methodological quality of RCTs in physical therapy [45]. This scale is comprised by 11 items that can be scored with one (yes) or zero points (no) based on its presence or absence in the study [79]. The total score on the PEDro scale is calculated by adding the points from items 2–11. The methodological quality of an RCT can be poor (3 points or less), moderate (4–5 points), good (6–8 points) and excellent (9–10 points) [26]. The interpretation of each item assesses the risk of bias.

The quality of evidence of the results of each meta‐analysis was analysed based on the GRADE Assessment (Grading of Recommendations Assessment, Development, and Evaluation) and from Meader's checklist [49, 83]. Five elements were used to quantify the quality of the evidence: individual risk of bias in each study, inconsistency, imprecision, presence of indirect evidence, and risk of publication bias in a common finding. The inconsistency was determined assessing the statistical heterogeneity in meta‐analyses (see statistical analysis section). Imprecision was estimated from the number of studies and participants included in each meta‐analysis (large, <5 studies and <100 participants; moderate, 10–5 studies and 300–100 participants; and low (>10 studies and >300 participants). Indirect evidence was confirmed when a results were obtained indirectly. Finally, the combination of these items allows the evidence to be classified as strong (if the results are robust and all items are met), moderate (the meaning and magnitude of the results could be susceptible to change when incorporating new studies), low (level low confidence) and very low (the results are very uncertain since 4 or more items are not met). The quality of evidence was downgraded one level for each item not fulfilled.

### Statistical analysis

Meta‐analysis was performed using *Comprehensive Meta‐Analysis version 4* (Biostat). Meta‐analysis was only performed when at least two comparisons (*k* = 2) per variable were available. The pooled effect size was estimated using Cohen's standardised mean difference (SMD) and its 95% confidence interval (95% CI) in a random‐effects model by Dersimonian and Laird [9, 14]. The interpretation of the effect size was carried out from the recent study of Kinney et al. [38], who propose the following interpretation of the effect size in rehabilitation studies: null (SMD 0), small (SMD 0.08–0.15), medium (SMD 0.19–0.36) and big (SMD > 0.4). The results of each meta‐analysis were displayed graphically using the forest plots [69]. The risk of publication bias in each meta‐analysis was analysed based on 3 elements: (1) the funnel plots, (2) the *p* for the Egger test, and (3) to trim‐and‐fill estimation [16, 17, 73, 75]. If the funnel plot was asymmetric and the *p* < 0.1 for Egger, and the variation in trim‐and‐fill estimation was >10%, the presence of publication bias is suggested. Finally, heterogeneity is calculated from the *p* for the chi‐square test (*p* < 0.1 indicates possible heterogeneity) and the Higgins degree of inconsistency (*I* ^2^). Heterogeneity can be null (*I* ^2^ 0%), low (*I* ^2^ 10%–25%), medium (*I* ^2^ 25%–50%) or large *I* ^2^ > 50%) [32].

As an additional statistical analysis, we carried out a sensitivity analysis using the one study removed method to analyze the contribution of each study to the overall effect of the meta‐analysis.

## RESULTS

### Study selection

**Figure 1:**
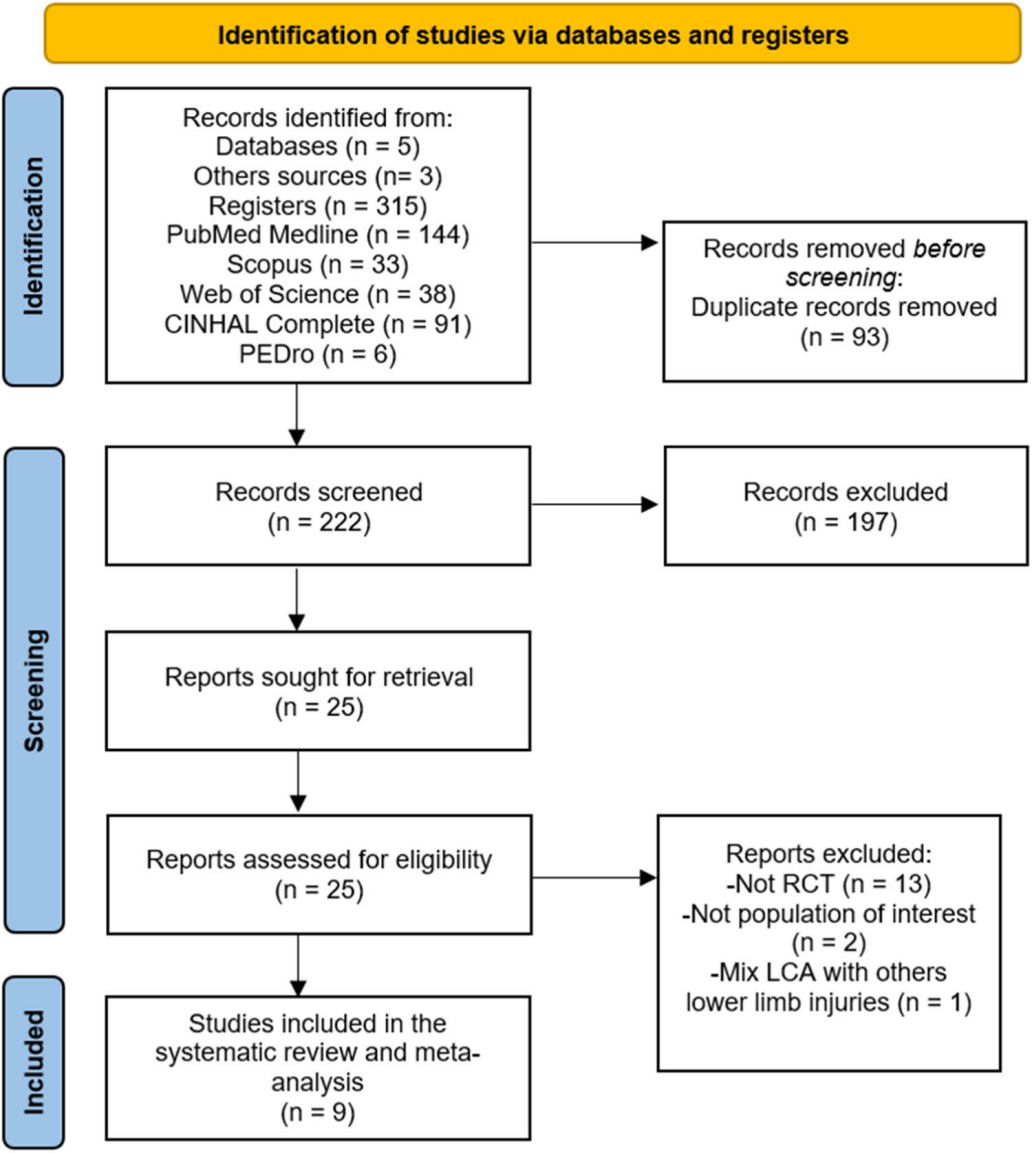
PRISMA flow diagram.

A total of 315 studies were retrieved, 312 from databases and three from other sources. After removing 93 studies as duplicates, title/abstract screening of 222 studies was performed. After this screening, 197 studies were removed for not being relevant showing a Kappa value for inter‐rater agreement of 0.91 (excellent agreement) for the title and abstract screening phase). Later, 16 studies were removed for not meeting the inclusion criteria (reasons in PRISMA flow diagram [Figure 1]). Finally, nine RCTs were included in this systematic review with meta‐analysis [1, 5, 7, 27, 35, 36, 43, 51, 54].

### Characteristics of the studies included

The RCTs included, carried out between 2012 and 2023 in Turkey [5, 35], Saudi Arabia [1, 54], China [43, 51], Malaysia [27], Iran [36] and Germany [7], provided data from 330 patients with ACL injury with a mean age of 26.96 ± 3.11 years (85.45% male). All studies provided data from patients who had recently undergone surgery for their injury, except one study which presented patients with a chronic ACL injury [54]. One hundred fifty‐nine patients (27.62 ± 3.28 years old) received VRBT and 171 patients (26.3 ± 2.96 years old) comprised the comparison group (conventional rehabilitation and physiotherapy techniques). In VRBT group, six studies [1, 5, 7, 35, 36, 43] used NIVR (Nintendo Wii Fit y Wii Balance Board, Nintendo Switch Ring Fit Adventure y GenuSport Knee Trainer); two studies [51, 54] used semi‐immersive VR (Dynstable); and a single study [27] used IVR (PlayStation‐VR). The VRBT protocols lasted between 3 and 12 weeks, with a frequency between 3 and 7 days per week, and sessions between 30 and 90 minutes. In six studies [1, 7, 27, 35, 43, 51] VRBT was performed as a therapeutic complement to a conventional rehabilitation or physiotherapy programme. All studies carried out an evaluation at the end of the treatment (immediate post‐intervention). Finally, four studies [1, 27, 35, 54] declared to receive external funding to conduct the research without reflecting a conflict of interest regarding funding. Table 1 shows the characteristics of the studies included.

**Table 1:** Characteristics of the studies included in the review.

Study	Sample	VRBT group		Control group		Variable y test	Qualitative findings in individual studies		
		Sample characteristics	VRBT intervention characteristics	Sample characteristics	Control intervention Characteristics		VRBT group	Control group	Inter‐group differences
Ahmed et al. [1] (Saudi Arabia) Non‐blinded RCT Setting: Alamal Hospital Funding: Yes.	30 Patients underwent ACL surgery	15 patients 25.5 ± 3.5 years old 15M:0F	NIVR device: Nintendo Wii Fit Application: 40 minutes per session, 3 times a week for 8 weeks. Combined with conventional rehabilitation.	15 patients 25.5 ± 3.5 years old 15M:0F	Therapeutic exercise in combination with conventional rehabilitation. Application: 40 min session, 3 times a week for 8 weeks.	Proprioception (isokinetic dynamometer)	Statistically significant increase (*p* < 0.001)	Statistically significant increase (*p* = 0.001)	Statistically significant differences favours VRBT group (*p* < 0.001)
Baltaci et al. [5] (Turkey) ECA doble ciego Setting: No especificado Funding: No	30 Patients underwent ACL surgery	15 Patients 28.6 ± 6.8 years old 15M:0F	NIVR device: Nintendo Wii Fit Application: 1 hour, 3 sessions per week for 12 weeks.	15 Patients 29.3 ± 5.7 years old 15M:0F	Conventional rehabilitation in progression from passive mobilisations, through closed kinetic chain potentiation, balance exercises and jogging.	Strength and proprioception (isokinetic dynamometer)	No significant improvement (*p* > 0.05)	No significant improvement (*p* > 0.05)	No statistically significant differences (*p* > 0.05)
						Dynamic balance (SEBT)	Significant improvement in anterior (*p* < 0.001) and postero‐medial (*p* = 0.002) CoP displacement	Significant improvement in posteromedial displacement (*p* = 0.038) of the CoP	No statistically significant differences (*p* > 0.05)
Karakoc, ZB et al. [35] (Turkey) Non‐blinded RCT Setting: Kartal Dr. Lütfi Kırdar Training and Research Hospital. Funding: Yes	22 Patients underwent ACL surgery	14 Patients 31 ± 8.41 years old 14M:0F	NIVR device: Nintendo Wii Balance Board. Application: 40 minutes, 3 times a week for 6 weeks. It is combined with conventional rehabilitation, and begins 45 minutes after the conventional part begins.	8 Patients 24 ± 5.9 years old 8M:0F	Conventional rehabilitation to reduce oedema, increase range of motion, strength and balance. Application: 45 minutes, 3 times a week for 6 weeks.	Pain (VAS)	Significant pain reduction (*p* = 0.005)	No significant improvement (*p* = 0.058)	No statistically significant differences (*p* > 0.64)
						Function (LEFS)	Significant increase in function (*p* = 0.002)	Significant increase in function (*p* = 0.012)	No statistically significant differences (*p* > 0.76)
Clausen, JD et al. [7] (Germany) Non‐blinded RCT Setting: Non‐specified health care clinic Funding: No	26 Patients underwent ACL surgery	14 Patients 24.8 ± 9.7 years old 6M:8F	NIVR device: GenuSport Knee Trainer. Application: 5 times a day for 3 weeks. Combined with conventional rehabilitation.	12 Patients 25.6 ± 6.4 years old 6 M:6F	Conventional rehabilitation programme based on knee mobilisations, strength exercises and gait training, for 3 weeks.	Strength (isokinetic dynamometer)	NR	NR	Statistically significant differences favours VRBT group (*p* < 0.02)
Gsangaya, MR et al. [27] (Malaysia) Non‐blinded RCT Setting: Non‐specified health care clinic Funding: Yes	30 Patients underwent ACL surgery	15 Patients 28.6 years old 13M:2F	IVR device: PlayStation‐VR Application: Used for 12 weeks as a complementary tool to a progressive conventional rehabilitation programme.	15 Patients 25.1 years old 10M:5F	Conventional rehabilitation programme similar to that of the TBRV group is used, but without using PlayStation‐VR	Pain (NPRS)	NR	NR	Statistically significant differences favours the VRBT group (*p* = 0.024)
						Knee flexion (goniometer)	NR	NR	No statistically significant differences (*p* = 0.517)
						Function (IKDC 2000)	NR	NR	Statistically significant differences favours VRBT group (*p* = 0.012)
						Dynamic balance (SEBT)	No significant improvement (*p* > 0.05) in any CoP displacement	No significant improvement (*p* > 0.05) in any CoP displacement	No statistically significant differences for anterior (*p* = 0.168), postero‐medial (*p* = 0.051) and postero‐lateral (*p* = 0.366) CoP displacement
Kazemnejad, A et al. [36] (Iran) Double‐blind RCT Setting: Non‐specified health care clinic Funding: No	20 Patients underwent ACL surgery	10 Patients 24.6 ± 3.7 years old 10M:0F	NIVR device: Nintendo Wii Fit Application: 90 minutes, 3 times a week for 4 weeks.	10 Patients 24.4 ± 3.2 years old 10M:0F	Conventional rehabilitation. Application: 90 minutes, 3 times a week for 4 weeks.	Pain (VAS)	Significant pain reduction (*p* < 0.001)	Significant pain reduction (*p* < 0.001)	Statistically significant differences favours VRBT group (*p* = 0.013)
						Knee flexion (goniometer)	Significant increase (*p* < 0.001)	Significant increase (*p* = 0.001)	Statistically significant differences favours VRBT group (*p* = 0.023)
						Dynamic balance (SEBT)	Significant improvement in anterior, postero‐medial and postero‐lateral (*p* < 0.001) CoP displacement	No significant improvement (*p* > 0.05) in any CoP displacement	Statistically significant differences favours VRBT group in anterior (*p* = 0.005), postero‐medial (*p* = 0.012) and postero‐lateral (*p* = 0.042) CoP displacement
Lu, YL et al. [43] (China) Single‐blind RCT Setting: Hospital de la Univesidad Médica de Wenzhou Funding: No	40 Patients underwent ACL surgery	20 Patients 30.9 ± 9.1 years old 16M:4F	NIVR device: Nintendo Switch Ring Fit Adventure. Application; 40 minutes, 3 times a week for 4 weeks. Combined with conventional rehabilitation.	20 Patients 29.1 ± 8.6 years old 15M:5F	Conventional rehabilitation with strength, balance and salgo exercises. Application: 40 minutes, 3 times a week for 4 weeks.	Pain (LKS, pain dimension)	No significant improvement (*p* = 0.163)	No significant improvement (*p* = 0.083)	No statistically significant differences (*p* = 0.679)
						Function (LKS)	Significant increase (*p* = 0.006)	Significant increase (*p* < 0.001)	Statistically significant differences favours VRBT group (*p* = 0.038)
						Strength (isokinetic dynamometer)	Significant increase (*p* < 0.001)	Significant increase (*p* < 0.001)	Statistically significant differences favours VRBT group (*p* = 0.019)
						Knee flexion (goniometer)	Significant increase (*p* < 0.001)	Significant increase (*p* < 0.001)	Statistically significant differences favours VRBT group (*p* = 0.036)
Nambi, G et al. [54] (Saudi Arabia) Double‐blind RCT Setting: Physical Therapy Department of Prince Sattam Bin Abdulaziz University Funding: Yes	60 Patients with ACL injury of chronic evolution	20 Patients 22.8 ± 1.3 years old 20M:0F	SIVR device: Dynstable Application: 15 minutes, twice a day, 6 days a week for 8 weeks.	GC_1_: 20 patients 21.9 ± 1.3 years old 20M:0F	Conventional rehabilitation based on stretching and strengthening. Application: 5 times a week for 4 weeks.	Pain (VAS)	Significant pain reduction (*p* = 0.001)	Significant pain reduction in both control groups (*p* = 0.001)	Statistically significant differences favours VRBT group (*p* = 0.001)
				GC_2_: 20 patients 22.6 ± 1.4 years old 20 V:0M	Static, dynamic and functional sensorimotor training. Application: 5 times a week for 4 weeks.	Function (WOMAC)	Significant increase (*p* = 0.001)	Significant increase in both control groups (*p* = 0.001)	Statistically significant differences favours VRBT group (*p* = 0.001)
Ming, S et al. [51] (China) Single‐blind RCT Setting: Jiaxing Hospital Funding: Yes.	72 Patients underwent ACL surgery	36 Patients 31.8 ± 2.7 years old 26M:10F	SIVR device: Dynstable Application: 15 minutes, 2 times a day, 6 days a week for 8 weeks. Combined with conventional rehabilitation	36 Patients 31.4 ± 2.4 years old 28M:8F	Conventional rehabilitation based on balance exercises. Application: 15 minutes, 2 times a day, 6 days a week for 8 weeks.	Knee flexion (goniometer)	Significant increase (*p* < 0.001)	Significant increase (*p* < 0.001)	Statistically significant differences favours VRBT group (*p* < 0.001)

Abbreviations: ACL, anterior cruciate ligament; CoP, centre of pressure; F, female; GC_1_, grupo control 1; GC_2_, grupo control 2; IKDC 2000, Subjective Knee Evaluation Form; IVR, immersive virtual reality; LEFS, Lower Extremity Functional Score; LKS, Lysholm Knee Joint Score; M, male; MSEBT, Modified Star Excursion Balance Test; NIVR, non‐immersive virtual reality; NPRS, Numeric Pain Rating Scale; NR, data non reported; RCT, randomised controlled trial; SIVR; semi‐immersive virtual reality; VAS, Visual Analogue Scale; VRBT, virtual reality‐based therapy; WOMAC, Western Ontario and McMaster Universities Arthritis Index.

### Assessment of methodological quality and risk of bias

The mean methodological quality was good (mean score of 6.88 ± 1.16 points in the PEDro scale), showing a medium risk of bias in individual. One study showed excellent methodological quality [54] (11% of the total) and the remaining 8 studies showed good methodological quality [1, 5, 7, 27, 35, 36, 43, 51] (89% of the total). Selectin bias was probable in two studies [43, 51] (item 3 was not met). Performance bias was present in six studies due to patients or therapists were not blinded [1, 7, 27, 35, 43, 51]. The evaluators were blinded in five studies (56% of the total) [5, 36, 43, 51, 54], so risk of detection bias has to be taken into account to generalise our findings. Table 2 shows the PEDro score of each included study.

**Table 2:** PEDro score of studies included in the review.

	Items												
Authorship	i1	i2	i3	i4	i5	i6	i7	i8	i9	i10	i11	Total	Quality
Ahmed et al. [1]	Yes	Yes	Yes	Yes	No	No	No	Yes	No	Yes	Yes	6/10	Good
Baltaci et al. [5]	Yes	Yes	Yes	Yes	No	Yes	Yes	Yes	No	Yes	Yes	8/10	Good
Karakoc, ZB et al. [35]	Yes	Yes	Yes	Yes	No	No	No	Yes	No	Yes	Yes	6/10	Good
Clausen, JD et al. [7]	Yes	Yes	Yes	Yes	No	No	No	Yes	Yes	Yes	Yes	7/10	Good
Gsangaya, MR et al. [27]	Yes	Yes	Yes	Yes	No	No	No	Yes	No	Yes	Yes	6/10	Good
Kazemnejad, A et al. [36]	Yes	Yes	Yes	Yes	Yes	No	Yes	Yes	No	Yes	Yes	8/10	Good
Lu, YL et al. [43]	Yes	Yes	No	Yes	No	No	Yes	Yes	No	Yes	Yes	6/10	Good
Nambi, G et al. [54]	Yes	Yes	Yes	Yes	Yes	No	Yes	Yes	Yes	Yes	Yes	9/10	Excellent
Ming, S et al. [51]	Yes	Yes	No	Yes	No	No	Yes	Yes	No	Yes	Yes	6/10	Good

*Note*: 1: Eligibility criteria; 2: Random allocation; 3: Concealed allocation; 4: Baseline comparability; 5: Blind subjects; 6: Blind therapists; 7: Blind assessors: 8. Adequate follow‐up; 9: Intention‐to‐treat analysis; 10: Between‐group comparisons; 11: Point estimates and variability. Eligibility criteria item does not contribute to total score.

Abbreviation: PEDro, Physiotherapy Evidence Database.

### Quantitative synthesis: Meta‐analyses

#### Pain

**Figure 2:**
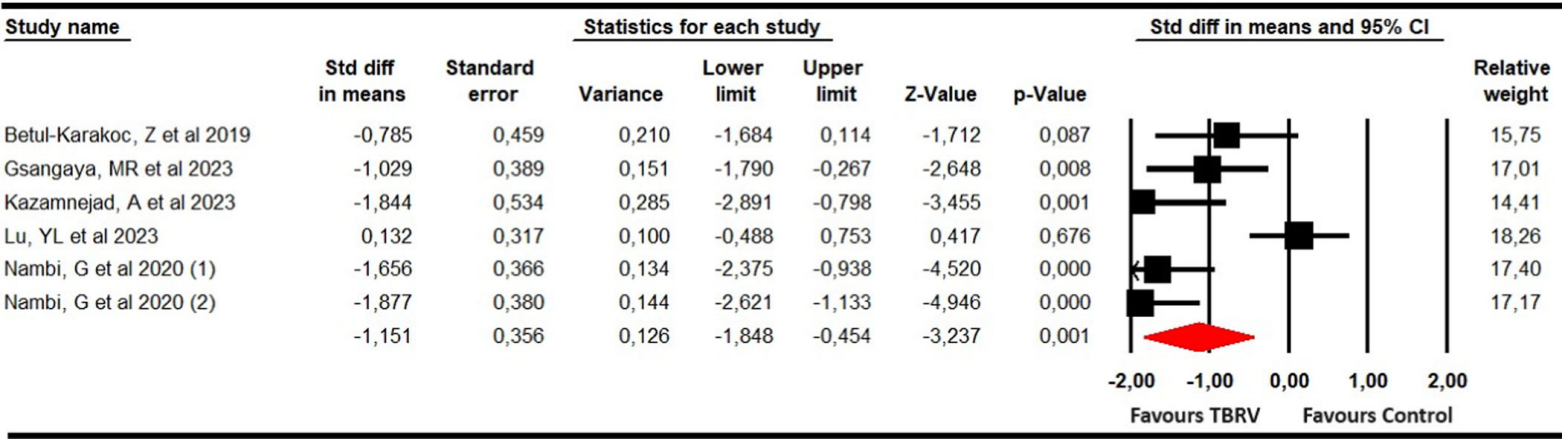
Forest plot for pain assessment. CI, confidence interval.

Pain was assessed with data from five studies [27, 35, 36, 43, 54] and 6 independent comparisons that provided data from 192 participants (32 participants per study) using the Visual Analogue Scale (VAS) and Numeric Pain Rating Scale (NPRS) for pain intensity and the pain dimension of the Lysholm Knee Joint Score (LKS). Our findings showed a large effect in reducing pain (SMD = −1.15; 95% CI −1.85 to −0.45; *p* = 0.001; *I* ^2^ = 0%; *Q* = 4.18; *df* = 5; *p* = 0.52) favours VRBT (Figure 2). Publication bias was sligthly present (*p* = 0.28), due to trim‐and‐fill estimation reported an adjusted effect of −1 (15% of variation respect original effect) (Supporting Information: Figure 1). This estimation suggests that the original effect would be overestimated by publication bias. Sensitivity analysis did not show variation.

#### Knee function

**Figure 3:**
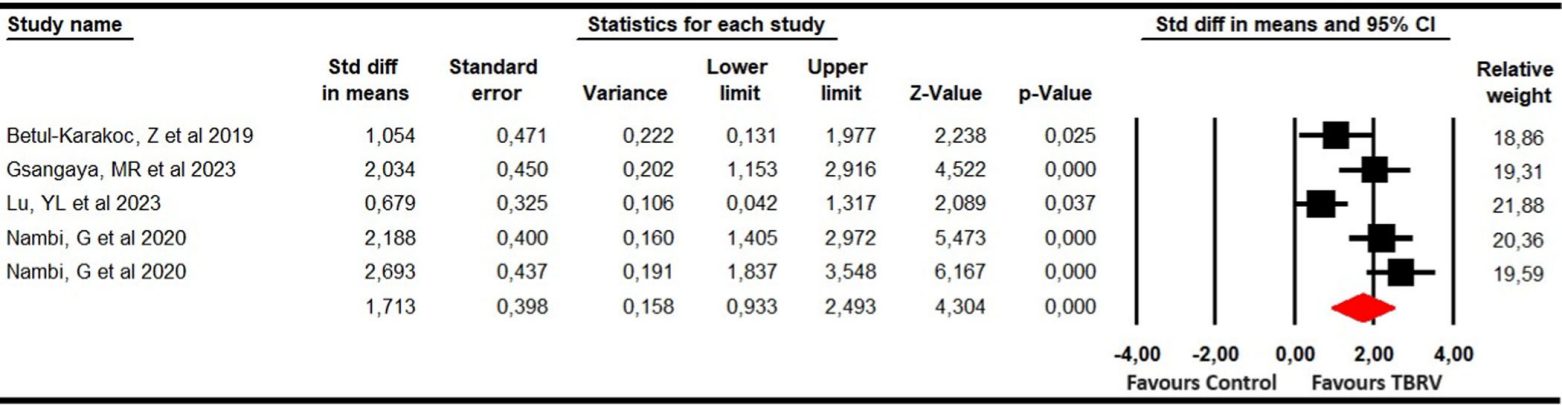
Forest plot for knee function. CI, confidence interval.

Knee function was evaluated with data from four studies [27, 35, 43, 54] with five independent comparisons that provided data from 172 participants (34.4 participants per study) using the Lower Extremity Functional Score (LEFS), Subjective Knee Evaluation Form (IKDC), LKS and the Western Ontario and McMaster Universities Arthritis Index (WOMAC). The meta‐analysis revealed a large effect in increasing knee function (SMD = 1.71; 95% CI 0.93–2.5; *p* < 0.001; *I* ^2^ = 0%; *Q* = 3.6; *df* = 4; *p* = 0.46) favours VRBT (Figure 3), without risk of publication bias. Sensitivity analysis did not show variation.

#### Knee strength

**Figure 4:**
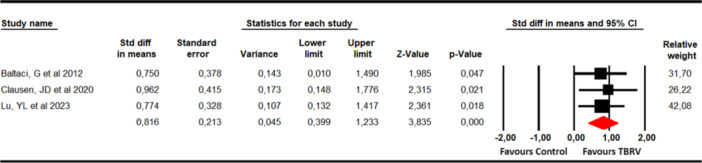
Forest plot for knee strength. CI, confidence interval.

On the one hand, knee strength was assessed with data from three studies [5, 7, 43] with three independent comparisons that provided data from 96 participants (32 participants per study) using isokinetic dynamometer. Our findings reported a large effect (SMD = 0.82; 95% CI 0.4–1.23; *p* < 0.001) in increase knee strength, favours VRBT (Figure 4), without heterogeneity (*I* ^2^ = 0%; *Q* = 0.17; *df* = 2; *p* = 0.92) and without risk of publication bias.

#### Knee proprioception

Knee proprioception was assessed through data from two studies with two independent comparisons [1, 5] and 60 participants (30 per study). No statistically significant differences were showed in increasing knee proprioception with VRBT (SMD = −1.64; 95% CI −5.46 to 2.17; *p* = 0.39) without heterogeneity (*I* ^2^ = 0%; *Q* = 0.13; *df* = 1; *p* = 0.72). Sensitivity analysis did not show changes.

#### Knee flexion ROM

**Figure 5:**
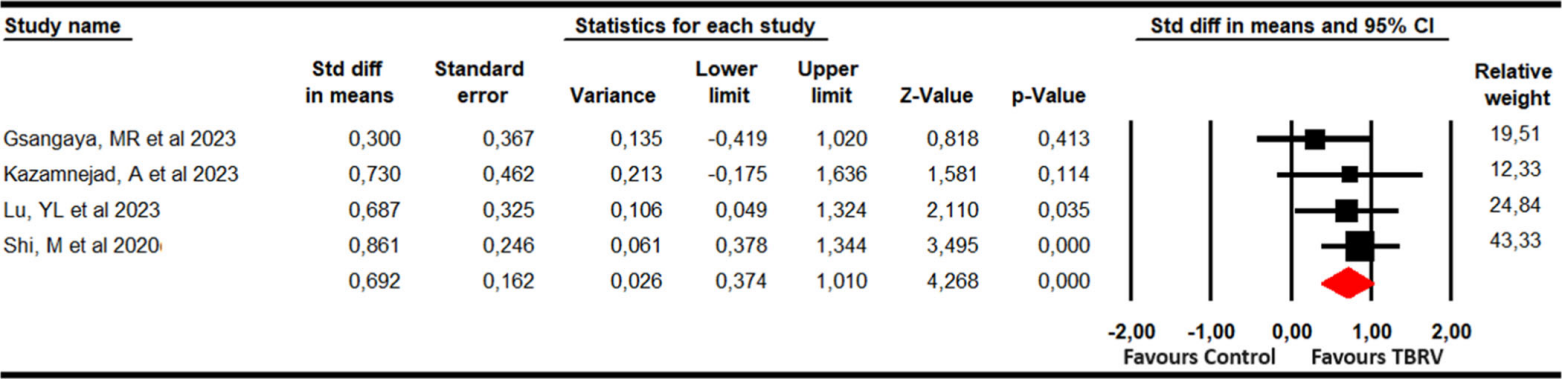
Forest plot for knee flexion ROM. CI, confidence interval; ROM, range of motion.

Knee ROM flexion was analysed with data from four studies [27, 36, 43, 51] with four independent comparisons that provided data from 162 participants (40.5 participants per study) using dynamometer and goniometer. Our results revealed a large effect in increasing knee flexion ROM (SMD = 0.7; 95% CI 0.37–1.01; *p* < 0.001; *I* ^2^ = 0%; *Q* = 1.61; *df* = 3; *p* = 0.65) favours VRBT (Figure 5), without risk of publication bias. No variations were reported in sensitivity analysis.

#### Dynamic balance

**Figure 6:**
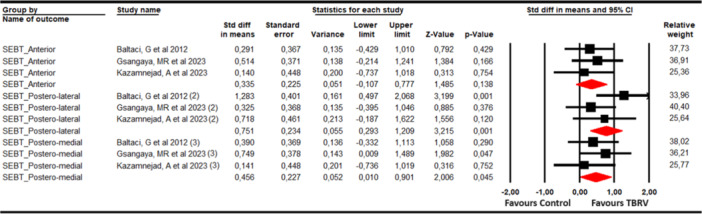
Forest plot for dynamic balance. CI, confidence interval.

The ability to maintain the dynamic balance using the affected leg was assessed using the Star Excursion Balance Test (SEBT) with data from three studies [5, 27, 36] with three independent comparisons. Three meta‐analyses were performed for each SEBT dimension, reporting statistically significant improvements favours VRBT in postero‐medial (SMD = 0.46; 95% CI 0.01–0.9; *p* = 0.045; *I* ^2^ = 15.1%; *Q* = 3.1; *df* = 2; *p* = 0.21) and postero‐lateral CoP excursion (SMD = 0.75; 95% CI 0.3–1.21; *p* = 0.001; *I* ^2^ = 0%; Q = 1.13; *df* = 2; *p* = 0.57), but not for anterior CoP excursion (SMD = 0.34; 95% CI −0.11 to 0.78; *p* = 0.138; *I* ^2^ = 0%; *Q *= 0.43; *df* = 2; *p* = 0.81) (Table 3, Figure 6). Risk of publication bias was found in postero‐medial (*p* = 0.11), in which adjusted effect size was 0.57, showing that the original effect was underestimated by the publication bias (Supporting Information: Figure 2). No variations were reported in sensitivity analysis.

## DISCUSSION

VRBT, that combines gamification with active tasks, is emerging as a rehabilitation tool in physiotherapy programmes of ACL injuries [13]. To date, the review of Fernandes et al 2022 did not show effectiveness of VRBT in the rehabilitation of ACL injuries [21]. Since these authors carried out the literature search (June, 2021), three new studies have been published in 2023 [27, 36, 43] and others two [51, 54], that meeting the inclusion criteria, were not included in that review, making necessary to perform our meta‐analysis that shows a variation compared to previous findings. Additionally, this meta‐analysis presents more homogeneous findings with major level of evidence comparing to Fernandes et al. [21] due to included more RCTs and participants (*n* = 9, four more than previous review; and 220 patients more than previous review); and did not combine RCTs studies with cases‐healthy control studies, like the previous reviews including the study of Gokeler et al. [25]. Therefore, the aim of our meta‐analysis was to update the literature search and retrieve all published evidence to evaluate the effectiveness of VRBT in the rehabilitation of ACL injuries. Our meta‐analysis showed that VRBT could be effective in reducing pain and increasing knee function, flexion ROM, proprioception and balance after ACL injuries. So, the present meta‐analysis shows more robust, generalisable and with major level of evidence findings, that increases the robustness of our conclusions, highlighting that VRBT is an effective tool to use in the rehabilitation of ACL injuries. The scientist evidence derived from this meta‐analysis about the effectiveness of VRBT the rehabilitation of ACL injuries will help clinicians and patients to cooperate together in “shared decision‐making” processes with the aim to find and choose the most adequate treatment according each patient [62], that reducing complications and sadness feeling, can increase the recovery during rehabilitation using VRBT.

Our meta‐analysis showed that VRBT is effective in reducing pain and increasing knee functional scores during ACL rehabilitation. These findings (including 3 RCTs more both variables) contradicts the results of Fernandes et al. [21] for these variables, providing clinical evidence to clinicians about the effectiveness of VR devices for reducing pain and increasing knee functional scores in rehabilitation programmes after ACL injuries [21]. Additionally, our findings agrees with others recent meta‐analyses [20, 24] that confirm that VRBT can be effective in reducing pain and increase knee function in others knee conditions, such as total knee replacement [20, 24]. Pain experienced after ACL injury or during ACL rehabilitation is related with the knee functional scores recovery, due to this unpleasant perception or discomfort does not allow you to perform some functional tasks or exercises [27]. Conventional physiotherapy uses passive therapies to reduce pain, such as electrotherapy, cryotherapy or manual therapy, but its effects seem only maintained in short‐time. Opposite, VRBT has emerged as an effective therapy in reducing pain in different musculoskeletal syndromes [67] basis on distraction and immersion phenomena. Distraction aims to divert the patient's attention from what they may perceive as unpleasant [76], causing external stimuli to compete with endogenous pain for our attentional resources [28, 67]. Distraction produces neurophysiological changes acting on different brain areas related to pain, such as primary and secondary somatosensory cortex, frontal gyrus, anterior cingulate cortex, insula, and left thalamus, left supramarginal gyrus and right posterior cerebellum, altering the pain perception [11, 67], and reducing it. Furthermore, positive emotions or feelings can increase the efficacy of distraction, and in this sense, VR‐based games allow to perform funny tasks in ludic environments that increase the motivation of the patient in the rehabilitation [37], and consequently can reduce the level of pain. The reduction of pain experienced after VR exposition and the possibility to perform functional and gamified tasks with customised conditions (intensity, time, level of difficulty and the selection of the virtual environment more appropriated or motivating for each patient) favours the immersion and adherence in the knee rehabilitation processes [41]. This adherence to rehabilitation is extremely important, especially in the athlete population, with evidence that adherence to treatment and its maintenance beyond three months represent one of the main predictors of return to play [68]. Adherence and motivation favours the repetitive practice of rehabilitative exercises that can be directly related with an improvement in knee functional scores.

Next, our meta‐analysis revealed that VRBT is effective in increasing knee strength and knee flexion ROM during ACL injury rehabilitation, but not in knee proprioception. The effectiveness of VRBT in increasing knee strength is an unprecedented finding in literature, due to Fernandes et al. [21] did not find statistically significant differences favours VRBT in this variable. It can be explained for two reasons, first the major number of RCTs included in the meta‐analysis of this variable (*n* = 3), and second because in the previous review, authors included a case‐control study with the healthy subjects in her meta‐analysis, could altering the truth effect of VRBT. The improvements in knee functional scores allow to training functional activities during more time and with more intensity, favoring the increase of muscle strength in lower limb. Several studies carried out in the last 5 years, coincide in that VRBT seems to be a useful tool to improve muscle strength in knee injuries [22, 33], and our meta‐analysis increases the number of knee pathologies, ACL injuries in this case, that may benefit from VRBT effects. Literature hypothesise that attentional focus could be one of the mechanisms involved in the increase of muscle after VRBT. Functional tasks performed with VRBT help patients shift their attentional focus from an internal focus on structure to an external focus on the various stimuli that VRBT provides. This external focus of attention not only helps reduce pain [82], but studies have also shown that it leads to greater strength gains compared to an internal focus [66]. Finally, this meta‐analysis is the first that evaluates the effect of VRBT on knee flexion ROM, and the positive findings are in line with other recent meta‐analysis about total knee replacement [20]. In 2018, one study showed that adding VRBT to conventional rehabilitation reduces two days the time spent in obtaining 90° of knee flexion ROM [34], and probably this finding can be extrapolated to patients involving in the first stages of the ACL rehabilitation.

In contrast to Fernandes et al. [21], this meta‐analysis, with data from 3 studies, reported that VRBT is effective in increasing dynamic balance, being able to reduce the excursion of the centre of pressure in postero‐medial and postero‐lateral directions. Due to ACL is a structure with large sensorimotor involvement to maintain the postural control [15], the multi‐sensory stimulation provided by VRBT may be responsible of this improvement in balance [42]. Compared to healthy controls, to assess postural balance using the SEBT in patients with ACL injury has showed improvements the postero‐medial and postero‐lateral CoP displacements, while in the anterior displacement these are very large moderate or null [12]. On the other hand, in studies that have found a significant reduction in the anterior displacement in the injured leg, when observing the non‐injured leg they have found significant differences in the posteromedial and posterolateral displacement but not in the anterior [30]. These results together suggest that postural and sensorimotor control demands are not as high from the ACL in anterior movement.

The present systematic review with meta‐analysis presents updated clinical contributions of interest for clinicians involved in the rehabilitation and recovery of ACL injuries. Our findings confirm that VR devices are a safe and effective therapeutic complement to conventional physiotherapy techniques in the rehabilitation of knee injuries, including ACL [24, 48]. To date, this is the meta‐analysis with the most complete literature search, retrieving the major number of studies, that must to be taken into account when VRBT was used in the rehabilitation of ACL injuries. VR devices allow to design safe, customised, ludic and virtual environments in clinics or at home (telerehabilitation [10, 23]) to training exercises or functional tasks with gamification that increase the adherence of the patient to the therapy, could maximise the effect of rehabilitation [63]. Furthermore, patients that perform exercises through VRBT, feel less fatigue during therapy favoring adherence and active participation [6, 78]. VRBT can be used since the early stages of rehabilitation being able to speed up the rehabilitation and the recovery [18]. Additionally, this adherence can be reinforced with the use of other novel technological implementations in the prehabilitation and rehabilitation after surgical ACL reconstruction, such as smartphone apps. These apps, *Orthopy app* for example, provide support to the patients during rehabilitation providing information about the management of possible complications, detailed and video‐guided explanations of exercises and self‐assessment tools of pain, function and quality of life, providing quick feedback of their own recovery progress [71]. The combined use of VRBT as complement in conventional therapy with smartphone apps will help to patients and clinicians to increase the quality of the therapy and follow‐up, facilitating the communication between them. One recent study showed that 5 years after ACL reconstruction no all patients experienced a satisfactory recovery. Pain, discomfort or stiffness are setbacks during rehabilitation that reduce that provides feelings of sadness and despair related with *“a past that will not come back”* [65]. On all patients, but specially in these, it is crucial to provide a continuous follow‐up and increase the positive feelings in them with the aim to enhance its motivation and the adherence with the therapy, being VR devices an excellent tool to get it.

Although our findings are relevant, some limitations must be taken into account. First, the low number of studies and participants included in each meta‐analysis was not major than 5, restricting the quality of evidence according GRADE statement and the generalisation of our findings. However, this meta‐analysis retrieved all evidence published to date, increasing the level of evidence than previous review. Regarding to the sex of patients involved, the majority were male, reducing the generalisation of female sex. Second, the risk of selection (related to an inadequate concealed allocation), performance and detection biases (related to the impossibility to blinding participants and therapists, and evaluators, respectively) in some of the RCTs included in the meta‐analysis downgraded the quality of evidence of the results and can alter the truth effect (accuracy) of the VRBT producing a possible overestimation of the findings [3, 4]. Third, the risk of publication bias advertised in the meta‐analysis of pain and dynamic balance suggested that the original pooled effect could be overestimated. Therefore, the adjusted pooled effect calculation using trim‐and‐fill estimation confirmed that the effect of VRBT on reducing pain should be slightly smaller (13%) without publication bias. Fourth, although the level of heterogeneity in meta‐analyses were inexistent or very low, the variability between in the modality of VR devices (immersive, semiimmersive or nom‐immersive) used in the studies included; the differences between protocol parameters (number of sessions, sessions per week and minutes per session); if VRBT was used as unique therapy or complement to conventional rehabilitation; and the differences in sample size between studies, can difficult the comparability between studies, reducing the generalisation of our findings. Finally, the studies included did not provide data to assess the effect of VRBT along time. Due to, our meta‐analysis did not provide information about whether the effect of VRBT will be sustained over time (follow‐up).

## CONCLUSION

The present systematic review with meta‐analysis has evaluated the effectiveness of VRBT in the recovery of ACL injury. VRBT emerges as a safe and effective therapy that can be included in physiotherapy rehabilitation programmes in reducing pain and improving knee function, strength, flexion ROM, and dynamic balance in patients with ACL injury. However, no statistically significant differences were found between VRBT and conventional rehabilitation in increasing knee proprioception. It is important to highlight that the number of RCTs published to date is low and it limits the quality of evidence and generalisation of these results. Besides, we encourage to the future researchers to perform new RCTs with lower level of biases, especially trying to conceal adequately the allocation and blinding therapists and all evaluators with the aim to obtain more robust results. Furthermore, is essential to increase the number of participants per study, especially female sex and homogenise the protocol application of VRBT with the aim to gain comparability between studies.

## AUTHOR CONTRIBUTIONS

*Conceptualization and design*: Irene Cortés‐Pérez, José María Desdentado‐Guillén, María Soledad Camacho‐Delgado, María del Rocío Ibancos‐Losada, Esteban Obrero‐Gaitán and Rafael Lomas‐Vega. − *Methodology*: Irene Cortés‐Pérez, Esteban Obrero‐Gaitán and Rafael Lomas‐Vega. *Literature search, study selection and data extraction*: Irene Cortés‐Pérez, José María Desdentado‐Guillén, María Soledad Camacho‐Delgado and Rafael Lomas‐Vega. − *Methodological quality and risk of bias assessment*: Irene Cortés‐Pérez, María del Rocío Ibancos‐Losada and Rafael Lomas‐Vega. − *Statistical analysis*: Irene Cortés‐Pérez and Esteban Obrero‐Gaitán. − *Writing the manuscript*: Irene Cortés‐Pérez, José María Desdentado‐Guillén and Esteban Obrero‐Gaitán. − *Revision and editing of the manuscript*: María Soledad Camacho‐Delgado, María del Rocío Ibancos‐Losada and Rafael Lomas‐Vega. − *Supervision*: Esteban Obrero‐Gaitán and Rafael Lomas‐Vega. − *Visualisation and validation*: Irene Cortés‐Pérez, José María Desdentado‐Guillén, María Soledad Camacho‐Delgado, María del Rocío Ibancos‐Losada, Esteban Obrero‐Gaitán and Rafael Lomas‐Vega. All authors provided critical feedback and helped shape the research, analysis and manuscript.

## CONFLICT OF INTEREST STATEMENT

The authors declare no conflict of interest.

## ETHICS STATEMENT

Not applicable.

## DECLARATIONS

PROSPERO Register id: CRD42023462985. Artificial Intelligence has not been used or consulted to write the manuscript.

## Supporting information

Supplementary information.

## Data Availability

The data that support the findings of this study are available on request from the corresponding author.
