# Supplementary material for: Virtual reality‐based therapy after anterior cruciate ligament injury effectively reduces pain and improves knee function, movement patterns, and dynamic balance: A systematic review and meta‐analysis
Source: Knee Surg Sports Traumatol Arthrosc. 2024 Sep 20;33(5):1736–53. doi: 10.1002/ksa.12477 (PMC12022836; doi:10.1002/ksa.12477)
Supplement: Supplementary file 1 — Supplementary information. [file KSA-33-1736-s001.docx]

**Supplementary Table 1.** Search strategy used in each database**.**

| **Database** | **Search strategy** |
| --- | --- |
| **PubMed Medline** | (Anterior Cruciate Ligament[mh] OR Anterior Cruciate Ligament*[tiab] OR Anterior Cruciate Ligament Reconstruction[tiab] OR Anterior Cruciate Ligament Injuries[mh] or Anterior Cruciate Ligament Injur*[tiab] OR Anterior Cruciate Ligament Rupture[tiab] OR ACL[tiab] or ACL injur*[tiab] or ACL tear*[tiab] or ACL rupture[tiab]) AND (Virtual Reality[mh] OR Virtual Reality[tiab] OR Virtual Reality Exposure Therapy[mh] OR Virtual Reality Exposure Therapy[tiab] OR Exergaming[mh] OR Exergam*[tiab] OR VR[tiab] OR “Wii”[tiab] OR “Play Station”[tiab] OR videogam*[tiab] OR “Nintendo”[tiab] OR immersive virtual reality[tiab] or Kinect[tiab] or simulator[tiab] or virtual environment[tiab] or serious games[tiab] or non inmersive virtual reality[tiab]) |
| **SCOPUS** | TITLE-ABS-KEY (“Anterior Cruciate Ligament” OR “Anterior Cruciate Ligament Reconstruction” OR “anterior cruciate ligament injuries” OR “Anterior Cruciate Ligament Rupture” OR “ACL” OR “ACL injure” OR “ACL tear” OR “ACL rupture”) AND TITLE-ABS-KEY (“Virtual Reality” OR “Virtual Reality Exposure Therapy” OR “Exergaming” OR “Exergame” OR “VR” OR “Wii” OR “Play Station” OR “videogame” OR “Nintendo” OR “immersive virtual reality” or “Kinect” OR “simulator” OR “virtual environment” OR “serious games” OR “non immersive virtual reality”) |
| **Web of Science** | Title (*Anterior Cruciate Ligament* OR *Anterior Cruciate Ligament Reconstruction* OR *anterior cruciate ligament injuries* OR *Anterior Cruciate Ligament Rupture* OR *ACL* or *ACL injure* or *ACL tear* or *ACL rupture*) AND Title (*Virtual Reality* OR *Virtual Reality Exposure Therapy* OR *Exergaming* OR *Exergame* OR *exergames* OR *Wii* OR *Play Station* OR *videogame* OR *Nintendo* OR *immersive virtual reality* or *Kinect* or *virtual environment* or *non immersive virtual reality*) |
| **CINAHL Complete** | AB (Anterior Cruciate Ligament OR Anterior Cruciate Ligament Reconstruction OR anterior cruciate ligament injuries OR Anterior Cruciate Ligament Rupture OR ACL or ACL injure or ACL tear or ACL rupture) AND AB (Virtual Reality OR Virtual Reality Exposure Therapy OR Exergaming OR Exergame OR exergames OR VR OR Wii OR Play Station OR videogame OR Nintendo OR immersive virtual reality OR Kinect OR simulator OR virtual environment OR serious games OR non immersive virtual reality) |
| **PEDro** | Anterior Cruciate Ligament AND virtual reality  Anterior Cruciate Ligament AND Wii  Anterior Cruciate Ligament AND Kinect |


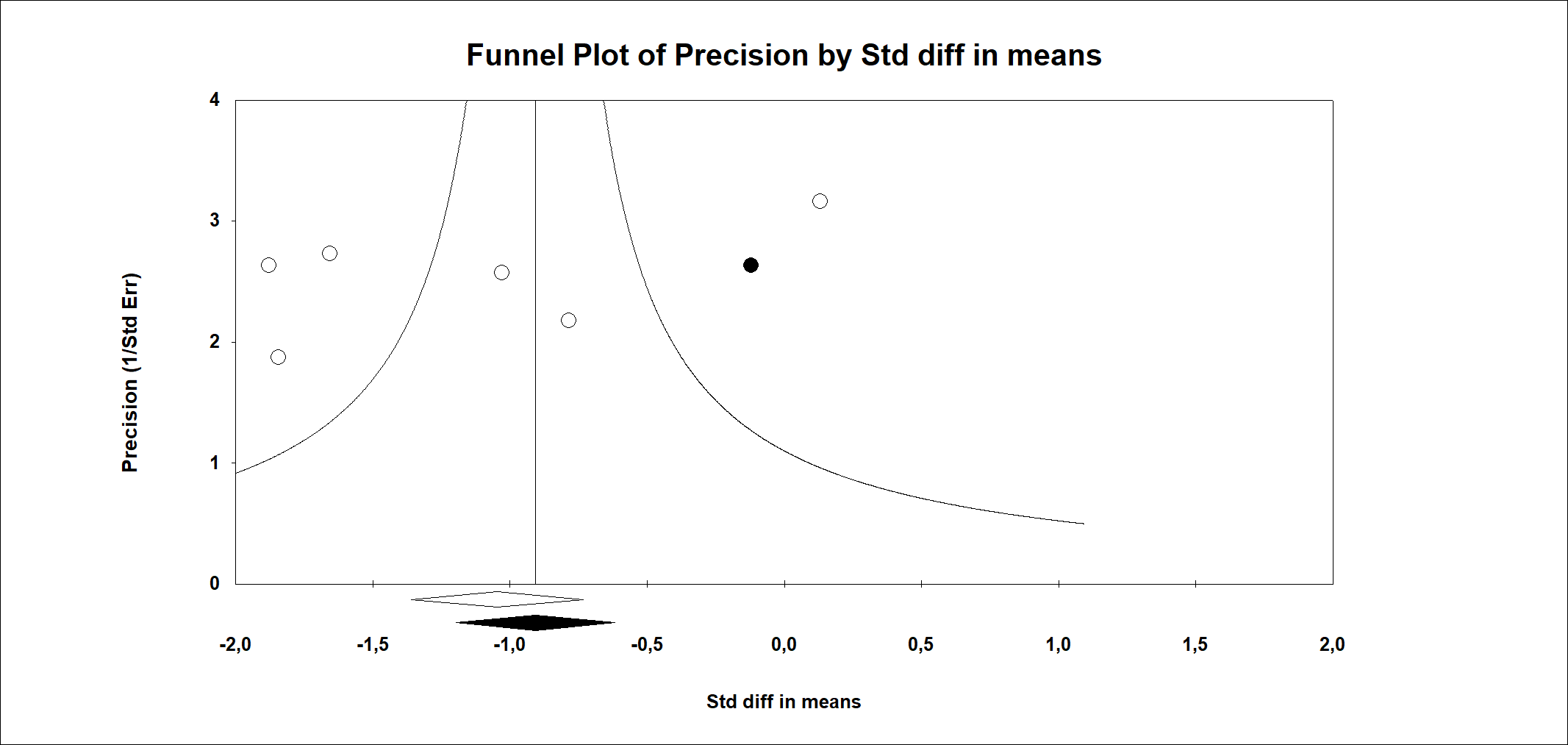


**Supplementary figure 1.** Funnel plot for pain


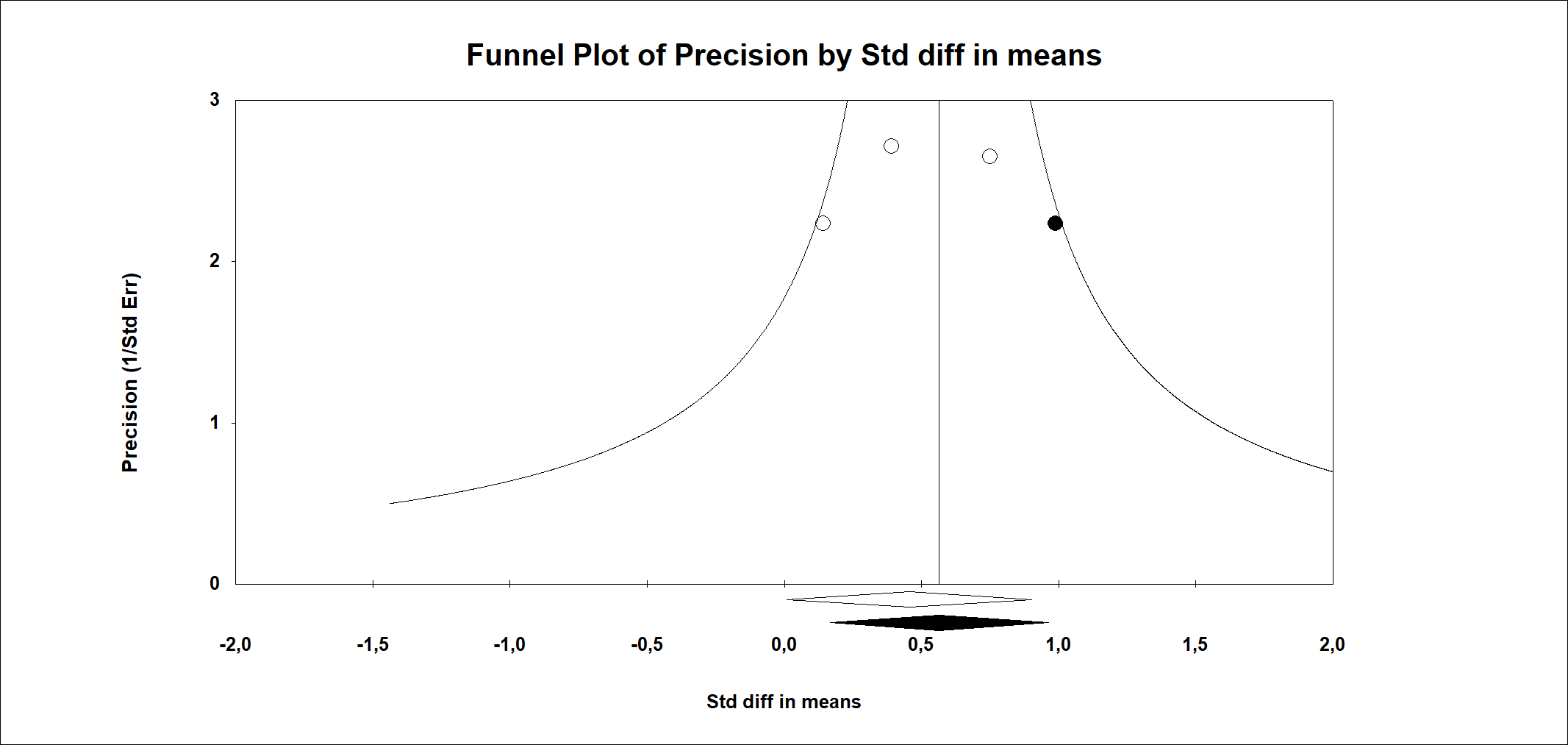


**Supplementary figure 2.** Funnel plot for dynamic balance (postero-medial CoP excursion)
